# Economic Burden of Alopecia Areata in the Kingdom of Saudi Arabia from a Societal Perspective: A Cost-of-Illness Analysis

**DOI:** 10.36469/001c.151864

**Published:** 2025-12-19

**Authors:** Khalidah Alenzi, Abdulrahman Alturaiki, Mohammad Fatani, Saad Alsogair, Arsalan Mohammad Saeed, Ayman Behiry, Ali Almomatin

**Affiliations:** 1 Tabuk Health Cluster, Riyadh, Saudi Arabia; 2 Ministry of the National Guard-Health Affairs, King Abdullah International Medical Research Center, King Saud bin Abdulaziz University for Health Sciences, Riyadh Saudi Arabia; 3 Dermatology Department Hera General Hospital, Makkah, Saudi Arabia; 4 Department of Dermatology Dr. Layla AlOnaizi Polyclinic, Khobar, Saudi Arabia; 5 Pfizer Scientific Technical Limited Company, Riyadh, Saudi Arabia

**Keywords:** economic burden, Saudi Arabia, alopecia areata, cost of illness

## Abstract

**Background:**

Alopecia areata (AA) is a chronic autoimmune condition characterized by non-scarring hair loss, with significant psychological, social, and economic implications. In Saudi Arabia, AA prevalence ranges from 2.3% to 13.8%, with early onset and strong familial predisposition. Despite its burden, data on the economic impact of AA in the region remain limited.

**Objectives:**

This study assessed the economic burden of AA in the Kingdom of Saudi Arabia from both public payer and societal perspectives, across varying disease severities including mild to moderate, severe, and refractory cases.

**Methods:**

A prevalence-based cost-of-illness model was developed using structured literature review, expert input, and primary data collection via questionnaires. The model estimated direct medical costs (drug acquisition, diagnostics, clinic visits), direct nonmedical costs (travel, accommodation), and indirect costs (productivity loss) over 1 year for mild to severe AA and 2 years for refractory cases. Cost data were sourced from official channels (National Unified Procurement Company and the Saudi Food and Drug Authority) and validated using Saudi Amazon and Al-Dawaa pharmacy platforms. Medical costs, including laboratory tests, diagnostic procedures, and all supportive therapies, were obtained from the Ministry of Health.

**Results:**

The average annual per-patient cost was SAR 20 703 for mild to moderate AA and SAR 76 957 for severe AA, translating into SAR 5.827 billion and SAR 3.352 billion total burden, respectively. Refractory AA cases incurred cumulative 2-year costs ranging from SAR 102 117 to SAR 167 615 per patient. Indirect costs, primarily due to productivity loss, were the dominant cost driver in mild to moderate AA and remained substantial across all severities.

**Discussion:**

Indirect costs, mainly productivity loss, drive the economic burden of AA in Saudi Arabia, with drug costs rising in severe cases. This pattern mirrors findings from global studies.

**Conclusions:**

AA imposes a significant financial burden in Saudi Arabia, driven largely by productivity losses and drug acquisition costs. These findings underscore the need for early diagnosis, standardized treatment protocols, and improved access to innovative therapies. Integrated care pathways and national registries are essential to optimize resource allocation and improve patient outcomes.

## BACKGROUND

Alopecia areata (AA) is a prevalent autoimmune disorder characterized by nonscarring hair loss, where the immune system attacks hair follicles, leading to partial or complete hair loss. It can manifest in varying severity, from small patches of hair loss to more extensive forms, such as alopecia totalis and alopecia universalis.^1–3^ Globally, AA affects approximately 2% of the population, with variations observed across different regions.^2^ In Saudi Arabia, studies have reported a prevalence ranging from 2.3% to 13.8%, indicating a higher occurrence compared with Western countries.^3,4^ The onset of AA in Saudi Arabia typically occurs between the ages of 11 and 30 years, with an average age at diagnosis of 18.6 years. Furthermore, a significant portion of patients (36%) report having a first-degree relative with AA, indicating a strong genetic predisposition to the condition.^4^

The impact of AA extends beyond physical manifestations, profoundly affecting patients’ psychological, social, and economic well-being. Studies have demonstrated that individuals with AA experience higher levels of depression and anxiety, and a diminished quality of life.^4–8^ This is further illustrated by findings from the Eastern Province and Makkah in Saudi Arabia, where a considerable proportion of patients reported significant psychological distress.^9,10^ Furthermore, the economic burden of AA is substantial, encompassing both direct medical costs (consultations, treatments, medications) and indirect costs such as productivity losses from absenteeism and presenteeism.^11^

The global economic burden of AA has been well-documented, with notable differences across regions. In the United Arab Emirates (UAE), patients with psychological comorbidities faced significantly higher healthcare utilization costs ($224.99 vs $103.70 for autoimmune-related disorders).^12^ In the United States, total annual medical costs for AA patients were substantially higher compared with matched controls, amounting to $8557 vs $6416, respectively. This difference was mainly due to the increase in ambulatory care costs ($3640 vs $2062) and pharmacy expenses ($3287 vs $1843), with immunologic agents significantly contributing to pharmacy costs.^11^ A similar trend was observed in Japan, where the annual cost of AA was estimated at ¥112.7 billion (US $857 million), with 78.2% of this burden attributed to productivity losses.^13^ In the Middle East, research on the economic implications of dermatological conditions like atopic dermatitis has highlighted significant healthcare expenditures and productivity losses.^14^ In the United Kingdom, the median total cost of services and products related to AA was £801.50 over 12 months, which corresponds to 3% of patients’ disposable income.^15^ In Romania, the cost for both mild and severe cases of AA in 2022 was estimated at €46.289 million and €1.89 million for adult and pediatric patients, respectively.^16^ A US claims-based study revealed that, in severe cases, the indirect costs associated with loss of work productivity surpassed direct medical expenses, with the total annual burden per patient estimated to exceed $10 000.^11^ However, despite the global evidence, data on the economic burden of AA specific to Saudi Arabia remains scarce. Given the chronic nature of AA and its potential progression from mild to severe and refractory stages, understanding its economic impact is crucial for effective healthcare resource allocation and policymaking. Therefore, this analysis aims to assess the economic burden of AA in Saudi Arabia from public payers and societal perspective, focusing on both adult and adolescent patients across varying severity levels, including mild to moderate, severe, and refractory cases. The evaluation considered the costs over a 1-year period for mild, moderate, and severe cases, and a 2-year period for refractory cases, accounting for both direct and indirect costs.

## METHODS

This study employed an Excel-based cost-of-illness model to evaluate the economic burden of AA in Saudi Arabia from both public payer and societal perspectives. The methodology included a structured literature review, a cost-of-illness analysis across public tertiary hospitals, and primary data collection through questionnaires. A critical literature review was conducted to inform model inputs by identifying available data on disease incidence, prevalence, and distribution (mild, moderate, severe cases), demographics (age, gender, comorbidities), diagnosis methodologies (physical exams, scores/tools, lab tests), and health events distribution (treatment approaches, nonpharmacological and indirect costs). These data were used to populate the model and estimate the economic burden of AA in Saudi Arabia. The literature review focused on gathering data from multiple sources regarding disease severity distribution across adult and adolescent populations, and comorbidities such as atopic dermatitis, depression, anxiety, and thyroid disorders. In addition, it provided insights into treatment approaches, the proportion of patients receiving conservative management, primary care visits, and the indirect costs of managing AA, including productivity losses. The review included searches across UpToDate, PubMed, Embase, and the Cochrane Library, as well as relevant gray literature from local sources. The search was primarily conducted in English, with Arabic-language articles reviewed when available. Key search terms included *alopecia, alopecia areata, economic burden, cost of illness, cost, Saudi Arabia, GCC* [Gulf Cooperation Council], *treatment, diagnosis, complications,* and *quality of life*.

Studies were selected based on relevance to the economic burden of AA, including those reporting direct medical costs, indirect costs, or out-of-pocket expenses. Eligible studies involved patients with confirmed AA diagnosis and included observational studies, clinical trials with economic endpoints, health economic evaluations, and cost-effectiveness analyses. Publications in English or Arabic were considered, with no restrictions on publication date or geographic region. Both adult and pediatric populations were included. Studies were excluded if they focused solely on clinical efficacy without economic outcomes, lacked full-text availability, addressed other types of alopecia without AA-specific data, or provided insufficient economic data. Case reports and series were excluded unless they reported economic outcomes, and duplicate publications or overlapping datasets were removed.

**Figure 1.**
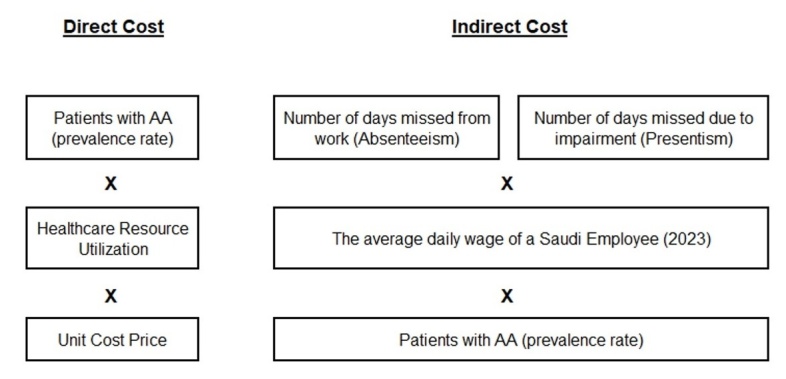
Research Design for Cost Estimation Model Abbreviation: AA, alopecia areata.

Following the literature review, a cost-of-illness study was conducted across public tertiary hospitals. To enhance the model’s accuracy, expert input was sought in areas where published data were limited, such as disease pattern distribution, demographic parameters, treatment approaches, complications, and indirect cost verification. Eleven experts were consulted, including dermatologists, clinical pharmacists, pharmacoeconomists, and pharmacy directors, and their opinions were incorporated to refine and validate model inputs. Structured questionnaires were then administered to fill in data gaps and validate assumptions, evaluating both direct and indirect costs associated with AA. Direct medical costs included expenditures related to drug acquisition, diagnostic and follow-up tests, medical resource utilization (clinic visits), treatment failure, complications, and nonpharmacological management. Medical costs, including laboratory tests, diagnostic procedures, and all supportive therapies, were obtained from the Self-Resources Department in the Ministry of Health. Direct nonmedical costs accounted for travel and accommodation expenses for adolescent patients requiring caregivers. Indirect costs were estimated based on work absenteeism and productivity loss for working patients, using the average daily wage in Saudi Arabia (**Figure 1**).

The model estimated per-patient costs across different disease states, categorized as mild to moderate, severe, and refractory AA. Patients with refractory AA were considered to have progressed from mild to moderate or severe disease over a 2-year period. The total economic burden was calculated using a prevalence-based approach, multiplying the per-patient costs by the estimated number of affected individuals. The data were retrieved through structured questionnaires administered to local experts and supplemented by published literature.^2,17–19^ Cost data were obtained from official sources such as the National Unified Procurement Company (NUPCO) and the Saudi Food and Drug Authority. In cases where official cost data were unavailable, pricing information was sourced from Saudi Amazon (2024) and validated using the online platform of a pharmacy chain in Saudi Arabia (Aldawaa), which offered comparable products in different formulations.

**Figure 2.**
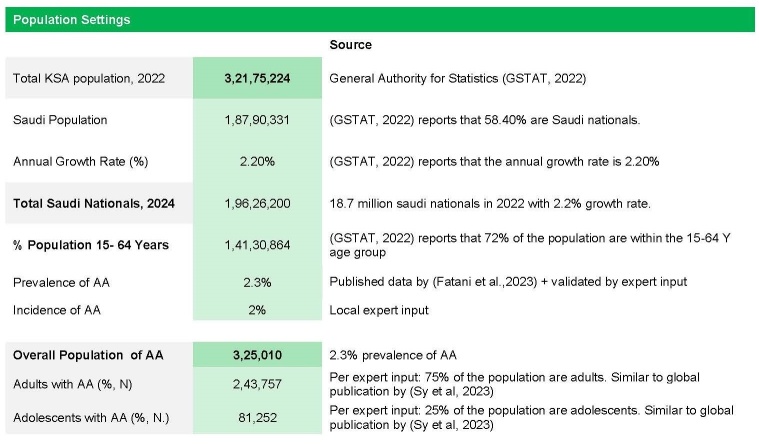
Population Settings Abbreviations: AA, alopecia areata; SAR, Saudi riyal.

The epidemiological data used in the model were extrapolated from published studies and validated by local experts. The prevalence and incidence rates of AA in Saudi Arabia were determined based on published literature and expert input.^2,17–19^ In the present model, the base-case prevalence of AA was set at 2.3%, consistent with published epidemiological studies.^17,18^ An annual incidence rate of 2% was also applied.^2^ To estimate the total Saudi population for 2024, population figures from the General Authority for Statistics (GASTAT, 2022) were adjusted using an annual growth rate of 2.2% as reported by GASTAT.^20^ The adjusted 2024 population was then multiplied by the 2.3% prevalence estimate to calculate the total number of individuals with AA. This total affected population was subsequently stratified into adolescents and adults, as illustrated in **Figure 2**. To account for population growth, adjustments were made using an annual growth rate reported in national statistics.

The distribution of AA severity was derived from published data and validated by expert input, with adults and adolescents demonstrating similar patterns. Patients were categorized into mild to moderate, severe, and refractory cases.^3,21,22^ Among adults, 89.4% of cases were classified as mild to moderate and 10.6% as severe, whereas among adolescents, 71.4% were mild to moderate and 28.6% were severe. Based on expert-defined criteria for refractory disease—failure of medication after 6 months or progression despite treatment—approximately 3% of adult cases and 2% of adolescent cases were considered refractory. Using a weighted average of 2.5%, refractory cases were further allocated according to disease progression, resulting in an estimated 7036 patients progressing from mild to moderate AA and 1089 patients progressing from severe AA.^3,21,22^

Healthcare resource utilization data were obtained from expert input and published sources.^13,23^ The model accounted for dermatologist consultations and follow-up visits, with the frequency of visits weighted based on treatment distribution and recommended clinical guidelines.^17,24^ The model assessed treatment patterns across different disease severities. Treatment categories included topical corticosteroids, intralesional steroids, and Janus kinase (JAK) inhibitors. Data on medication use and associated side effects were sourced from published literature and expert reports.^21^

Direct nonmedical costs were estimated by considering travel and accommodation expenses for patients and caregivers, particularly for adolescent cases. The experts reported varying percentages of patients who require travel to attend their appointments, with the average calculated at 21.6% for patients who are covered for travel and accommodation costs. Specifically, the costs associated with travel were SAR 500 for flights per person and SAR 500 for accommodation per person. These values were derived from expert input from business centers and were incorporated into the model for the estimation of direct nonmedical costs.

Indirect costs were calculated based on productivity loss due to work absenteeism and presenteeism. The impact of AA on work productivity varied by disease severity. The loss of productivity was estimated to be 13.9% for mild to moderate disease and 31.4% for severe disease. The experts reported absenteeism as 4 days per year for mild to moderate cases and 8 days per year for severe cases, primarily due to attending appointments. These values were also derived from expert input. The wage loss was estimated using the Saudi Workers Monthly Average Wage of SAR 10 238, as reported by GASTAT, leading to an average daily wage of SAR 465.38 based on 22 working days per month. These parameters were incorporated to calculate the total indirect costs due to absenteeism and productivity loss across the disease severity spectrum. The total economic burden was estimated by summing direct and indirect costs to assess the financial impact of AA on the healthcare system and affected individuals in Saudi Arabia.

## RESULTS

### Total Costs for Mild to Moderate AA

**Figure 3.**
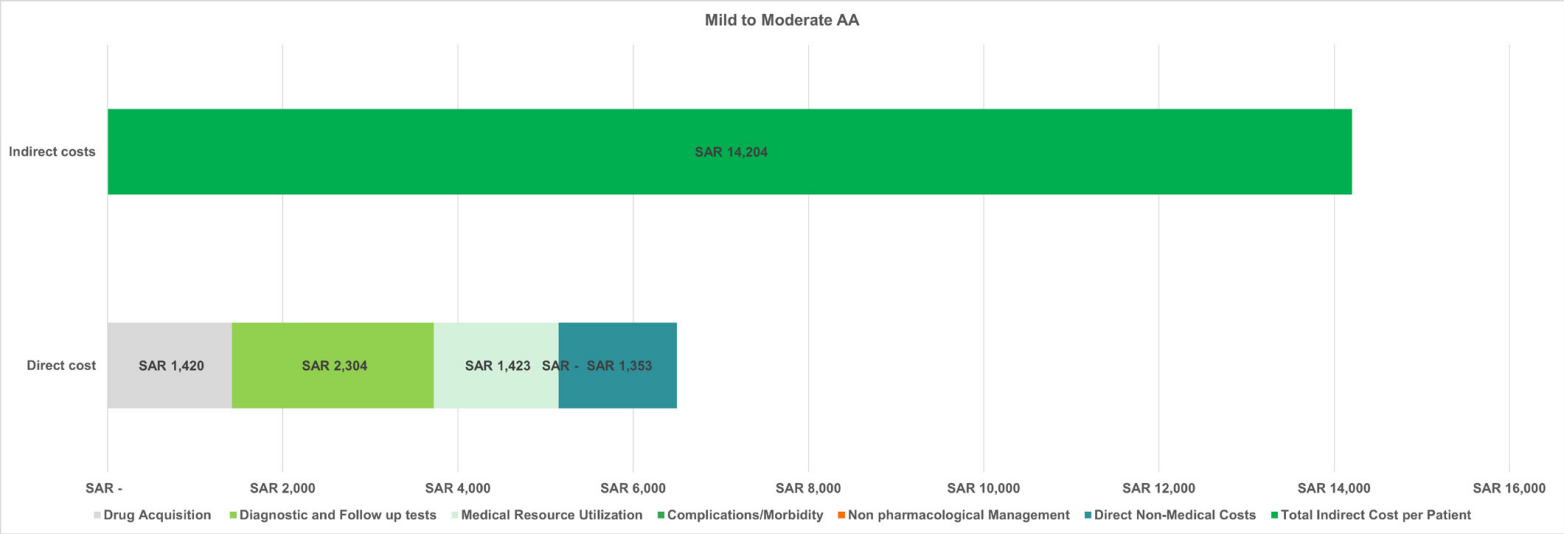
Per-Patient Cost Breakdown for Mild to Moderate AA Abbreviations: AA, alopecia areata; SAR, Saudi riyal.

The average per-patient total cost over 1 year for mild to moderate AA was SAR 20 703.30 ($5520.26), with the overall cost for all patients amounting to SAR 5.827 billion ($1.55 billion). The total per-patient cost was calculated by summing all direct and indirect costs. The overall costs were determined by multiplying the per-patient cost by the total number of patients in this disease category (**Figure 3**), which highlights that the indirect costs are the primary cost driver, accounting for nearly 70% of the total expense.

### Total Costs for Severe AA

**Figure 4.**
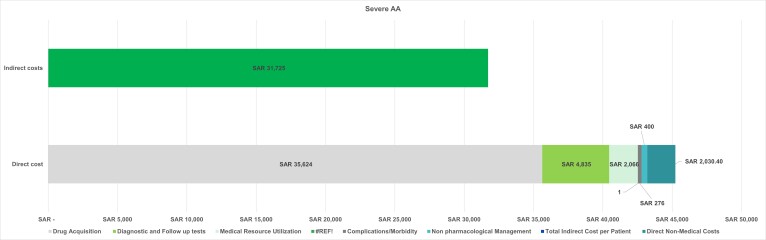
Per-Patient Cost Breakdown for Severe AA Abbreviations: AA, alopecia areata; SAR, Saudi riyal.

The average per-patient total cost in 1 year for severe AA was SAR 76 957.01 ($20 519.55), resulting in an overall cost of SAR 3.352 billion ($0.89 billion) for all patients. The total per-patient cost was determined by summing all direct and indirect costs. The overall costs were calculated by multiplying the per-patient cost by the total number of patients with this condition (**Figure 4**). This breakdown reveals that indirect costs comprise 41% of the overall cost, while direct costs account for 59%, with drug acquisition being the dominant cost driver, making up 46% of the overall cost and 79% of the direct costs. Notably, the main cost driver for drug acquisition was off-label treatments, as the approved therapies are not yet reimbursed.

### Total Costs for Refractory AA

**Total costs for refractory AA progressing from mild to moderate AA**: The total per-patient cost in the second year was determined by combining all direct and indirect costs. Meanwhile, the cumulative 2-year cost was calculated by adding the cost for mild to moderate AA in the first year to the cost for refractory AA in the second year. The overall costs were computed by multiplying the cumulative per-patient cost by the total number of patients in this disease state.

**Figure 5.**
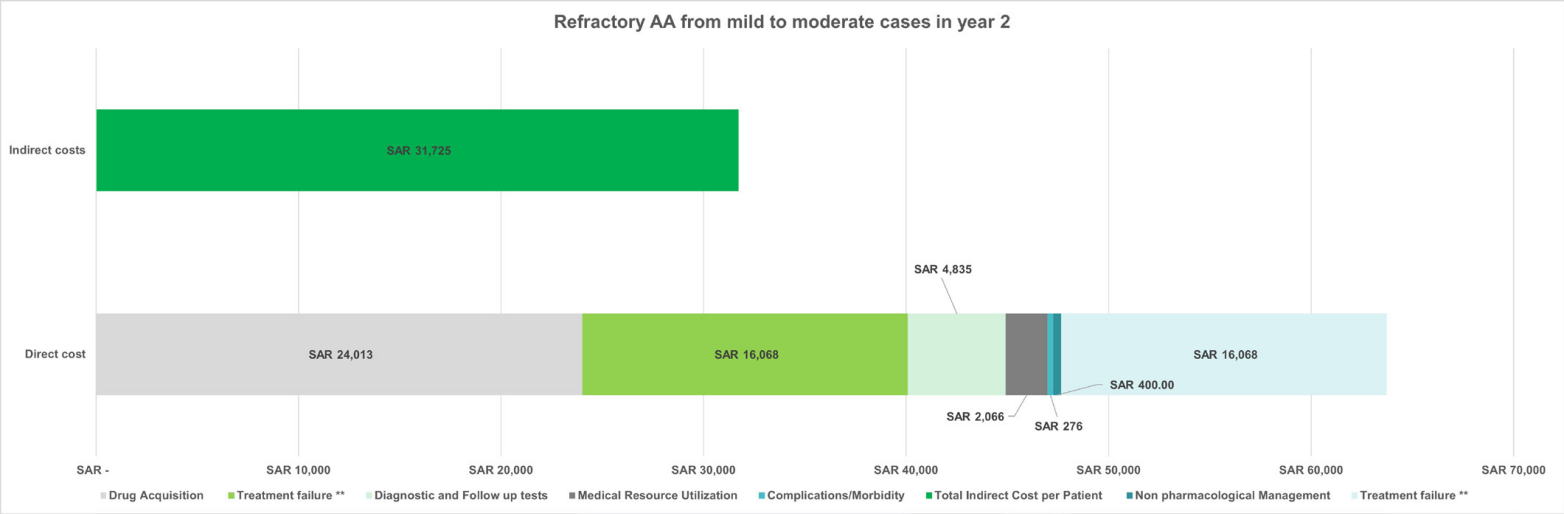
Per-Patient Cost Breakdown for Refractory AA from Mild to Moderate AA Abbreviations: AA, alopecia areata; SAR, Saudi riyal. **The cost was calculated for 6 months on new JAK inhibitor + 6 months on second JAK inhibitor.

The average total cost per patient in the second year was SAR 81 413.88 ($21 707.92), contributing to a cumulative 2-year cost of SAR 102 117.18 (27 228.18), with a total cost of SAR 718 million ($191.48 million) for all patients (**Figure 5**). This breakdown illustrates the per-patient cost analysis for refractory AA transitioning from mild to moderate severity, showing that the indirect costs constituted 39% of the total expenses, while direct costs comprised 61%. Specifically, drug acquisition and treatment failure costs accounted for 29% and 20% of the overall expenses, respectively, which amounts to 48% and 32% of the total direct costs, respectively.

**Total costs for refractory AA progressing from severe AA**: The total per-patient cost in year 2 was determined by combining all direct and indirect costs. The cumulative 2-year cost was obtained by summing the cost for severe AA in year 1 and the cost for refractory AA in year two. Overall costs were calculated by multiplying the cumulative per-patient cost by the total number of patients in this condition.

**Figure 6.**
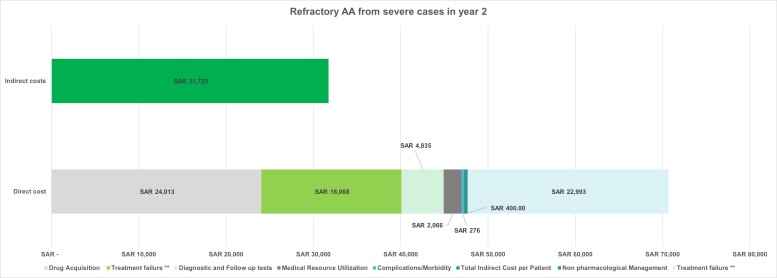
Per-Patient Cost Breakdown for Refractory AA from Severe AA Abbreviations: AA, alopecia areata; SAR, Saudi riyal. **The cost was calculated for 6 months on new JAK inhibitor + 6 months on second JAK inhibitor.

The average total cost per patient for refractory AA from severe AA in year 2 was SAR 90 658.39 ($24 172.84), leading to a cumulative 2-year cost of SAR 167 615.40 ($44 692.40), and an overall cost of SAR 182 million ($48.54 million) for all patients. (**Figure 6**). This breakdown demonstrates that the indirect costs accounted for 35% of the overall cost, while direct costs represented 65%. Drug acquisition and treatment failure costs contributed 29% and 25%, respectively, to the overall cost; these figures also made up 45% and 39% of the total direct costs, respectively.

### One-Way Sensitivity Analysis

To assess the robustness of the results, one-way sensitivity analysis was conducted by varying the input parameters by ±10%. The analysis for mild to moderate AA revealed that indirect costs were the most sensitive parameter. In severe AA, the most sensitive parameter was drug acquisition, followed by indirect costs. For refractory AA progressing from mild to moderate, indirect costs were the most sensitive, with drug acquisition and treatment failure being the next most impactful. Similarly, for refractory AA progressing from severe AA, indirect costs were the most sensitive parameter, followed by drug acquisition and treatment failure (**Supplementary Tables S1-S4**).

## DISCUSSION

To our knowledge, this is the first study to comprehensively evaluate the economic burden of AA from a societal perspective in Saudi Arabia, incorporating both direct medical expenditures and indirect productivity losses across disease severities. We estimated the average annual per-patient cost at SAR 20 703 ($5520.18) for mild to moderate AA and SAR 76 957 ($20 519.55) for severe AA, translating into SAR 5.8 billion ($1.55 billion) and SAR 3.35 billion ($0.89 billion) total burden, respectively. Indirect costs, such as lost wages and reduced on-the-job productivity accounted for nearly 70% of total costs in mild to moderate AA and 41% in severe AA, reflecting the substantial impact on the working-age population. These findings underscore that, beyond clinical management, AA imposes a heavy societal cost through work disability and diminished quality of life.

Baricitinib, a selective and reversible JAK 1/2 inhibitor, was recently approved by the US Food and Drug Administration,^25,26^ European Medicines Agency,^27,28^ and the Saudi Food and Drug Authority^17,29^ for severe AA in adults based on pivotal phase 3 BRAVE studies showing continued improvement over 52 weeks.^25,30^

At the time of our analysis, baricitinib was the only orally administered, regulator-approved therapy for severe AA in adults available in Saudi Arabia, while other systemic options were used off label.^17^ Despite approval, clinical uptake in Saudi Arabia has been gradual, as observed in a Saudi budget impact analysis that projected the market share of baricitinib rising only to ~3% by year 5, from SAR 5 836 616 to SAR 7 473 138 ($1 556 255.39 to $1 992 612.03),^31^ reflecting a slow integration into prescribing and payer formularies. Nevertheless, even modest uptake may reduce reliance on off-label immunosuppressants if accompanied by broader reimbursement and physician adoption. Our model similarly identified drug acquisition, particularly JAK inhibitors, as a key driver of direct costs (46% in severe AA; up to 29% in refractory cases), reinforcing the need for cost-effectiveness evaluations alongside budget impact studies.

In the current practice, however, off-label immunosuppressants and corticosteroids remain widely used.^31^ In a survey of physicians practicing in Taif, topical corticosteroids or minoxidil combined with systemic vitamins and minerals were the preferred choices, with response rates of 90% in localized AA and 40% in diffuse forms.^32^ Regional data mirror this pattern where an observational study across Egypt, Lebanon, Saudi Arabia, and the UAE found that potent topical corticosteroids were prescribed in 92% of mild to moderate and 78% of severe cases, while systemic immunosuppressants, calcineurin inhibitors, prostaglandin analogues, and phototherapy were used less frequently, often with variable efficacy, frequent relapse, and limited patient satisfaction.^21^ In moderate to severe disease, treatment remained dominated by corticosteroid-based strategies such as topical (78%), oral (67%), and intralesional (65%) along with topical minoxidil (70%) and topical calcineurin inhibitors (35%).^21^ Use of off-label JAK inhibitors was relatively uncommon (9%-15%), largely due to limited availability and lack of reimbursement.^21^ Across the region, awareness of investigational drugs was restricted, with only one-third of dermatologists reporting familiarity.^21^ Importantly, dermatologists identified persistent unmet needs, including long-term disease control (62%), improved efficacy (53%), faster onset of action (52%), and better safety profiles (51%).^21^

These patterns highlight the multifaceted challenges of off-label therapy in Saudi Arabia. A central concern is the lack of robust, AA-specific randomized evidence supporting sustained remission, with existing guidelines noting inconsistent responses and high relapse rates.^17,31^ Long-term safety further complicates their use as methotrexate and cyclosporine are associated with hepatotoxicity, nephrotoxicity, hypertension, and immunosuppression, requiring intensive monitoring, while prolonged systemic corticosteroid therapy carries risks of adrenal suppression and metabolic complications.^33–35^ In addition, the absence of standardized national protocols contributes to significant practice variation, increasing both coordination demands and financial burden for patients and payers. Finally, reimbursement gaps compound these issues, as off-label therapies are rarely covered by insurance, creating out-of-pocket costs and limiting access.^17,31^ Collectively, these challenges reinforce the limitations of off-label practice and strengthen the case for expanding access to approved, targeted therapies in Saudi Arabia.

At the regional level, AA places a significant burden on dermatologists and healthcare systems.^31^ Physician surveys across the Middle East, including Saudi Arabia, report persistent reliance on topical and intralesional corticosteroids, limited access to advanced therapies, and lack of standardized treatment guidelines.^21^ In the UAE, claims analyses further show that comorbidities, especially autoimmune and psychiatric disorders, drive substantially higher healthcare resource utilization.^12^ These findings align with our results, where comorbidities contributed significantly to indirect costs, and underscore the importance of integrated psychological support and standardized care pathways.

Globally, the burden of AA is substantial, with the Global Burden of Disease Study 2019 reporting an age-standardized disability-adjusted life-year rate of 7.51 per 100 000, disproportionately affecting young adults and females in high-income countries.^31,36^ A retrospective claims analysis highlighted that ambulatory and pharmacy services were the primary contributors to increased economic burden. Notably, patients with severe forms of AA, such as alopecia totalis or universalis, incurred even higher costs, underscoring the financial impact of disease severity. These insights emphasize the importance of early intervention and effective management strategies to mitigate the escalating costs associated with AA. Ingrassia et al conducted a survey study revealing that individuals with alopecia often face substantial out-of-pocket expenses for treatments and concealment strategies, such as over-the-counter products and camouflaging agents.^37^ These financial burdens were reported to affect patients’ ability to afford basic items of daily living, highlighting the profound economic and psychosocial impact of alopecia.^37^ The study further emphasizes the lack of insurance coverage for alopecia-related costs, suggesting a pressing need for policy reforms to alleviate the financial strain on affected individuals.^37^ In Germany, Austria, and Switzerland, individuals with AA reported an average annual out-of-pocket expenditure of €1248, primarily on hair replacement products and cosmetics.^38^ In Japan, the national economic burden was estimated at ¥112.7 billion, with 78.2% attributed to productivity losses.^13^ These international benchmarks confirm the patterns observed in Saudi Arabia, namely, that indirect costs are often higher than direct medical costs and that disease severity markedly escalates total burden.

Given the high share of indirect costs identified in our model, developing integrated care pathways could play a critical role in reducing the overall burden of AA in Saudi Arabia. Primary care services should function as the first point of triage, enabling early recognition of disease and ensuring timely referral to specialists according to Severity of ALopecia Tool (SALT) severity scores and patient-reported impact.^17^ Within specialist dermatology clinics, multidisciplinary collaboration is essential: pharmacists can oversee medication reconciliation, assess potential drug-drug interactions, and establish laboratory monitoring schedules for agents such as methotrexate, cyclosporine, or JAK inhibitors; psychologists and psychiatrists can provide screening and cognitive–behavioral therapy to address anxiety, depression, and body-image distress; and medical social workers can assist patients in navigating health coverage and reimbursement barriers.^7,39–46^ To further improve efficiency and consistency of care, step-care algorithms should be formally documented and aligned with the 2023 Saudi consensus guidelines, reducing unwarranted variation in clinical practice.^17^ In parallel, participation in national registries under the Ministry of Health would allow systematic collection of data on disease epidemiology, comorbidities, treatment outcomes, and safety signals, thereby strengthening evidence-based decision-making at both clinical and policy levels.^47^

The findings of this study underscore the need for a multifaceted approach to managing AA in Saudi Arabia. Healthcare policies should consider the inclusion of AA in reimbursement schemes, recognizing its impact beyond cosmetic concerns. Additionally, there is a pressing need to develop national registries and standardized treatment protocols to track epidemiology, resource use, and outcomes, and develop consensus guidelines to standardize care across regions. Furthermore, public awareness campaigns are essential to reduce stigma and encourage early intervention, which can mitigate both the clinical and economic burdens of AA. Integrating psychological support and addressing comorbidities within treatment plans can also enhance patient outcomes and reduce associated costs.

This study is subject to several limitations. The data utilized were based on available records, which may not capture all aspects of the disease burden, such as over-the-counter treatments and alternative therapies. Additionally, the analysis was conducted from the perspective of a public hospital payer, which may not provide a complete picture of the total societal costs. This perspective may overlook expenses incurred by patients and private insurers, resulting in an underestimation of the overall societal burden. Furthermore, the indirect costs were estimated using average wages reported by GASTAT; however, this approach may not account for variations across different regions, occupations, or genders. The model also did not include intangible costs, such as reduced quality of life, psychological distress, and the stigma associated with AA. These factors are significant but challenging to quantify in monetary terms. Furthermore, a significant portion of the data was supplemented by key opinion leader inputs to fill in gaps where literature data were unavailable. While these expert inputs were invaluable, they are inherently based on the perspectives of the experts and may not always reflect the precise, real-world distribution of costs, making these figures less than 100% accurate. Another limitation of the study is the lack of stratification of results by adult vs pediatric populations. The analysis of indirect costs did not fully capture caregiver burden for the pediatric population, which was considered only for 25% of adolescent cases. A breakdown by age group and a more detailed assessment of caregiver burden would have provided a more comprehensive understanding of the societal impact of AA.

## CONCLUSIONS

This study provides the first comprehensive assessment of the economic burden of AA in Saudi Arabia, highlighting both the direct medical and indirect societal costs associated with varying disease severities. Our findings show that AA imposes a significant financial strain, with indirect costs, particularly due to productivity loss, accounting for a substantial portion of the total economic impact. Notably, the cost burden increases with disease progression leading to financial burden on both patients and the healthcare system. These results are consistent with regional and global data, reinforcing the importance of early diagnosis, equitable access to effective treatments, and integrated care approaches. The cost of treatment, including high expenses associated with off-label use of JAK inhibitors, further contributes to the financial burden. Our study also points to the need for policy-level interventions to alleviate this burden. Addressing the economic and psychosocial impact of AA requires coordinated national strategies, such as establishing treatment guidelines, incorporating AA into reimbursement schemes, and improving public and healthcare provider awareness. Future research should focus on cost-effectiveness evaluations of emerging therapies and the long-term benefits of comprehensive disease management to support evidence-based policy decisions.

### Disclosures

A.M.B. is an employee of Pfizer and holds stock or stock options. A.M.S. was an employee of Pfizer Saudi Arabia at the time of this study.

## Supplementary Material

Online Supplementary Material
