# Supplementary material for: Economic Burden of Alopecia Areata in the Kingdom of Saudi Arabia from a Societal Perspective: A Cost-of-Illness Analysis
Source: J Health Econ Outcomes Res. 2025 Dec 19;12(2):253–61. doi: 10.36469/001c.151864 (PMC12718539; doi:10.36469/001c.151864)
Supplement: Online Supplementary Material [file jheor_2025_12_2_151864_322999.pdf]

Journal of Health Economics and Outcomes Research

Online Supplementary Material

Economic Burden of Alopecia Areata in the Kingdom of Saudi Arabia from a Societal Perspective: A Cost-of-Illness Analysis. *JHEOR*. 2025;12(2):253-261. doi:10.36469/jheor.2025.151864

Figure S1: Tornado Chart of the One-Way Sensitivity Analysis (±10%) of Per-Patient Costs for Mild to Moderate Alopecia Areata

Figure S2: Tornado Chart of the One-Way Sensitivity Analysis (±10%) of Per-Patient Costs for Severe Alopecia Areata

Figure S3: Tornado Chart of the One-Way Sensitivity Analysis (±10%) of Per-Patient Costs for Refractory Alopecia Areata from Mild to Moderate

Figure S4: Tornado Chart of the One-Way Sensitivity Analysis (±10%) of Per-Patient Costs for Refractory Alopecia Areata from Severe

This supplementary material has been provided by the authors to give readers additional information about their work.

This is an open-access article distributed under the terms of the Creative Commons Attribution 4.0 International License (CCBY-4.0). View this license's legal deed at http://creativecommons.org/licenses/by/4.0 and legal code at http://creativecommons.org/licenses/by/4.0/legalcode for more information.

Figure S1. Tornado Chart of the One-Way Sensitivity Analysis (±10%) of Per-Patient Costs for Mild to Moderate Alopecia Areata

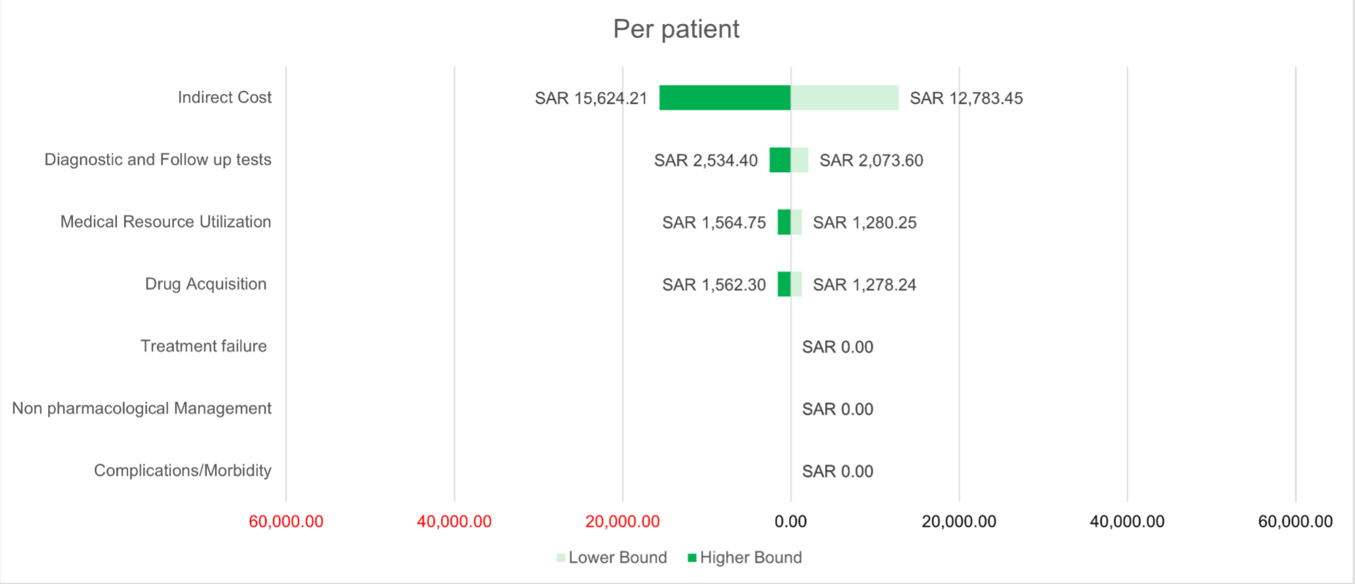

Abbreviation: SAR, Saudi riyal.

Table S2. Tornado Chart of the One-Way Sensitivity Analysis (±10%) of Per-Patient Costs for Severe Alopecia Areata

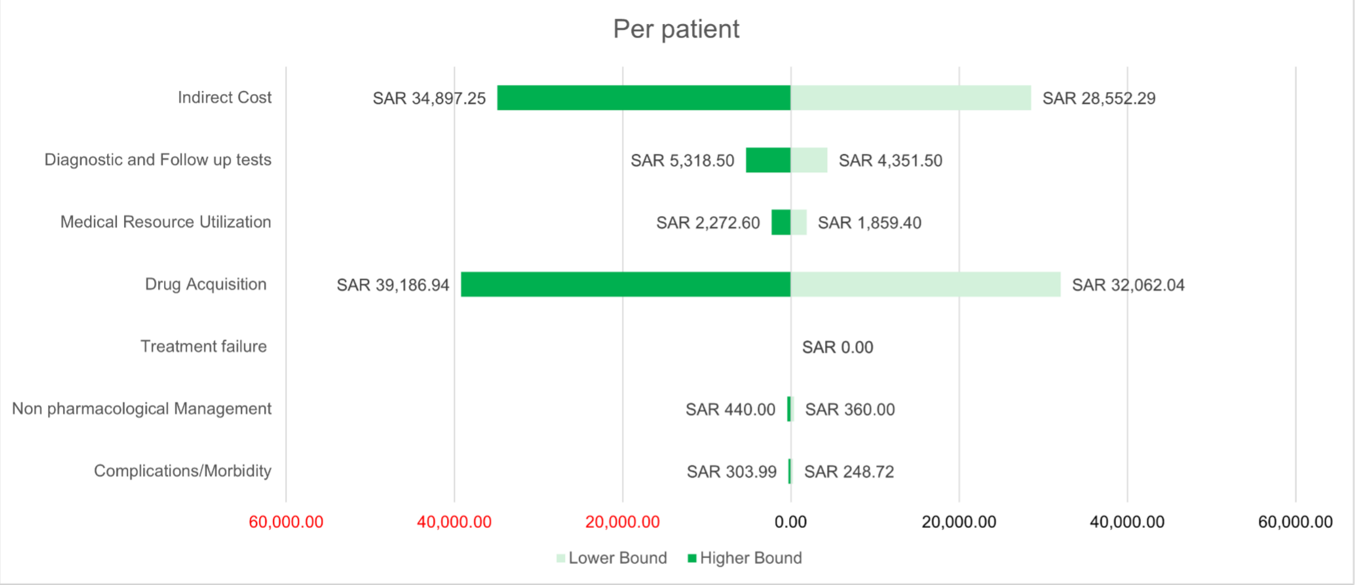

Abbreviation: SAR, Saudi riyal.

Table S3. Tornado Chart of the One-Way Sensitivity Analysis (±10%) of Per-Patient Costs for Refractory Alopecia Areata from Mild to Moderate

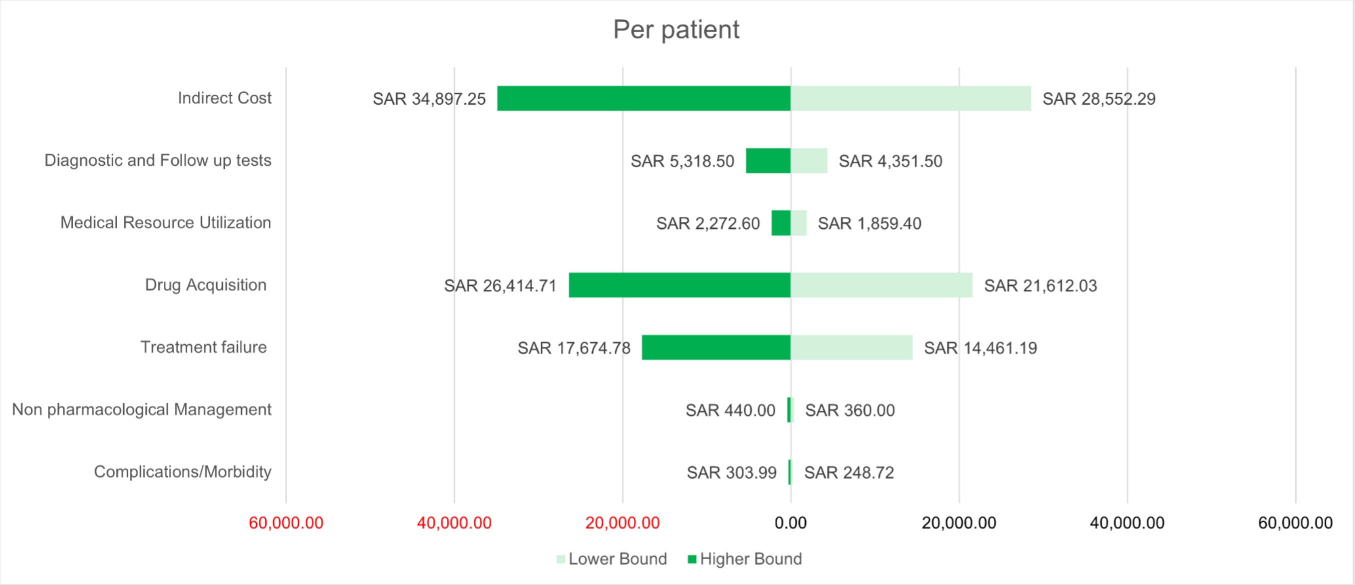

Abbreviation: SAR, Saudi riyal.

Table S4. Tornado Chart of the One-Way Sensitivity Analysis (±10%) of Per-Patient Costs for Refractory Alopecia Areata from Severe

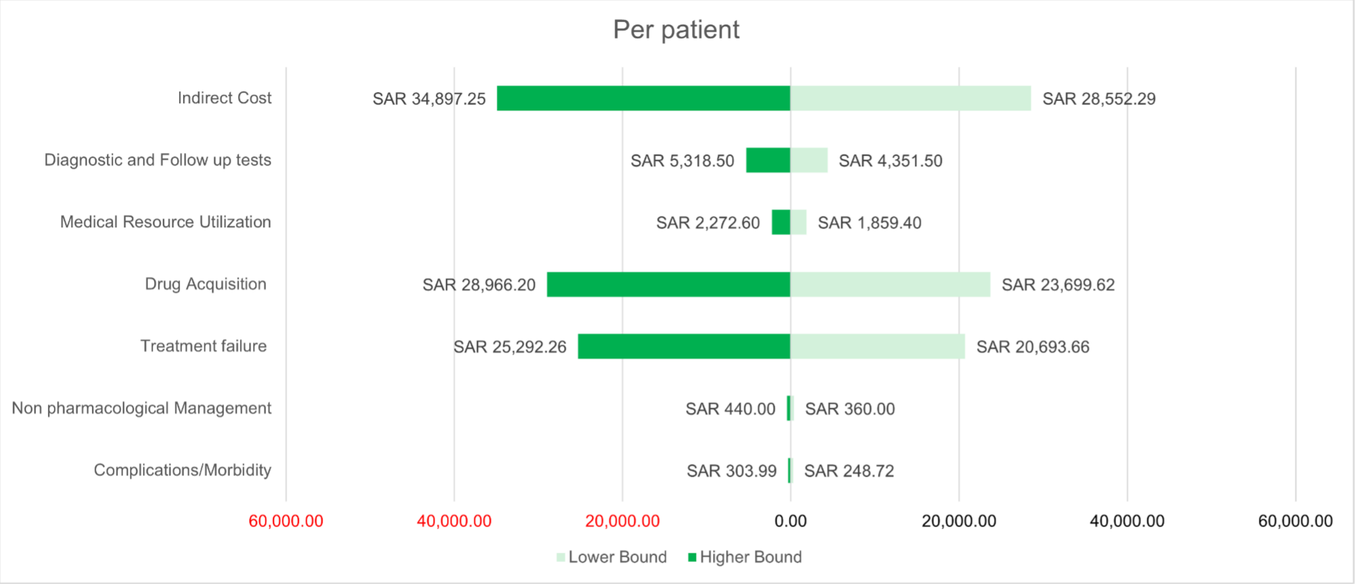

Abbreviation: SAR, Saudi riyal.
